# Two scales to measure suicide prevention communication among Latino parents: how do U.S.-born and foreign-born parents compare?

**DOI:** 10.3389/fpsyg.2025.1600765

**Published:** 2025-10-27

**Authors:** Tatiana Villarreal-Otálora, Tyler Collete, Jane McPherson

**Affiliations:** ^1^Wellstar College of Health and Human Services, Kennesaw State University, Kennesaw, GA, United States; ^2^Department of Psychological Sciences, Kennesaw State University, Kennesaw, GA, United States; ^3^School of Social Work, University of Georgia, Athens, GA, United States

**Keywords:** suicide, prevention, adolescents, Latino, parents, caregivers, scales, self-efficacy

## Abstract

**Introduction:**

This study validates two adapted versions of the Parental Suicide Prevention Communication Self-Efficacy scale (PSPCSE) for Latino parents of adolescents in the U.S.: the PSPCSE-Lat-E for predominately English speakers and the PSPCSE-Lat-S for Spanish speakers. These scales aim to measure suicide-related prevention communication self-efficacy (SPCSE) among Latino parents.

**Methods:**

Researchers adapted the PSPCSE to address lethal-means safety and incorporated culturally sensitive language to devise the PSPCSE-Lat-E (11-items), which was then translated and back-translated to create the PSPCSE-Lat-S (11-items). Both scales were included in a 60-question survey that also gathered demographic data, prevention programming preferences, familism levels, and suicide literacy. Participants were recruited through online panels and social media, resulting in responses from 220 foreign-born and 414 U.S.-born Latino parents (*N* = 634). An exploratory factor analysis examined the PSPCSE-Lat-E and PSPCSE-Lat-S psychometric properties.

**Results:**

Analysis (*N* = 634) revealed distinct structures: a two-factor structure for the English (48.8% variance, *ω*=.88) versus a three-factor for the Spanish (51.6% variance, *ω*=.82) in the rotated models. PSPCSE-Lat-E respondents reported higher confidence in discussing suicide content, while PSPCSE-Lat-S respondents showed greater comfort with emotional support items. PSPCSE-Lat-S suicide items demonstrated exceptional discrimination despite lower means, suggesting heightened cultural sensitivity among these parents.

**Discussion:**

Findings indicate that the PSPCSE-Lat-E and PSPCSE-Lat-S are reliable and valid tools for assessing Latino parent’s SPCSE. These scales can now be used in clinical and community settings, offering significant implications for health providers and future research. Differences in prevention tasks between foreign-born and U.S.-born Latino parents necessitate tailored interventions.

## Introduction

The Centers for Disease Control and Prevention’s (CDC, 2022) *Suicide Prevention Resource for Action* outlines the best evidence-based approaches to reduce suicide in the United States. These approaches are based on seven strategies delineated by the CDC to substantially reduce suicide, including (a) strengthening economic supports, (b) creating protective environments, (c) improving access and delivery of suicide care; (d) promoting healthy connections; (e) teaching coping and problem-solving skills; (f) identifying and supporting people at risk; and (g) lessening harms and preventing future risk (Stone et al., 2017). When it comes to preventing suicide among adolescents, these multi-level approaches and strategies impact and, in many instances, are dependent on parental^1^ involvement. The quality of this involvement depends upon parents’ belief in their ability to engage in appropriate suicide-related prevention communication with their adolescents (Bandura, 1977). This belief—also known as self-efficacy—is the perception of the capability to carry out a behavior effectively (Bandura, 1977).

Suicide is the third leading cause of death among Latino adolescents in the U.S. (American Psychiatric Association, 2022). Suicidal ideation and behaviors among Latino adolescents have been steadily growing since 2007 (Brewer et al., 2022) and have surpassed rates of suicidal ideation and behaviors among Black and White adolescents (Gomez et al., 2011). Parents may be the first to be exposed to their children’s suicide warning signs, for example, increased signs of anxiety or making jokes about suicide. If parents perceive themselves as incapable of responding helpfully to these signs, there is a high probability that they will not respond at all (Glatz et al., 2024; Jones and Prinz, 2005; Klassen, 2004). Such probability is heightened among Latino parents, who often view mental illness-related topics as stigmatizing and anxiety-provoking (Brewer et al., 2022).

Additional environmental, personal, and behavioral factors may also influence Latino parents’ self-efficacy with suicide-related prevention communication. Research suggests that multiple variables—including living in collectivistic versus individualistic countries, country of origin, level of acculturation, and previous exposure to the parental behavior of focus—all influence the strength of parental self-efficacy (Boruszak-Kiziukiewicz and Kmita, 2020; Buchanan et al., 2024; Wittkowski et al., 2017). For example, among Latino parents residing in the U.S., those who were U.S.-born may have more experience (personally or vicariously) with mental health-related topics, including suicide-related prevention communication, than those who were foreign-born.^2^ Findings from the National Research Center on Hispanic Children and Families reveal that the prevalence of mental health disorders is significantly lower among foreign-born Latino parents as compared to U.S.-born Latino parents (29% compared to 51%; Canady, 2022). Although foreign-born Latino parents may have lower exposure to mental health disorders, alarmingly, their adolescent children experience significantly higher rates of depressive disorders (28% higher) compared to youth of U.S.-born parents (Potochnick et al., 2024).

Given the variability among the different sub-populations of Latino parents in the U.S. and the potential impacts on their adolescents’ mental health, it is crucial to have tools that specifically explore suicide-related prevention communication self-efficacy and tailor prevention efforts for Latino sub-populations. Thus, to provide such tools, the authors of this article slightly adapted and validated the Parental Suicide Prevention Communication Self-Efficacy (PSPCSE) scale (Czyz et al., 2018) for use with Latino parents of adolescent children in the U.S., in both English and Spanish. Although the original PSPCSE scale was developed in English, it was validated with a predominantly non-Latino, Caucasian sample, with only 2.5% identifying as Latina (Czyz et al., 2018); thus, re-validation was necessary to ensure cultural relevance and psychometric robustness for Latino parents, whose experiences, language use, and cultural values may influence how they interpret and respond to suicide prevention communication items. The scales, collectively known as PSPCSE-Lat, comprise both English (PSPCSE-Lat-E) and Spanish (PSPCSE-Lat-S) versions. In this paper, the authors also analyze the data collected from the validation study to explore possible similarities and differences in suicide-related prevention communication between foreign-born and U.S.-born Latino parents of adolescents living in the U.S.

## Literature review

### Parental self-efficacy and suicide prevention communication with adolescents

Research findings consistently suggest that strong parental self-efficacy across many areas of parenting enhances young people’s overall functioning (c.f., Dzeidee Schaff et al., 2024; Matthews et al., 2022; Nicolas et al., 2020). For example, schools where initiatives to strengthen parental self-efficacy have been implemented see less disruptive behavior among adolescent students (Matthews et al., 2022; Resnik et al., 2023). Such behavioral impact is related to the positive effects of higher levels of parental self-efficacy on adolescent emotional regulation and self-esteem (Buchanan et al., 2022; Jones and Prinz, 2005; Nicolas et al., 2020), which are two pivotal protective factors against suicide behaviors among Latino youth (Santiago et al., 2017). Previous findings suggest that when a teenager struggles with suicidal ideation, high parental self-efficacy increases the likelihood that parents will initiate a life-saving conversation with their youth (Czyz et al., 2018).

This concept of self-efficacy is rooted in Bandura’s (1977, 1982) Social Cognitive Theory of behavioral change. The theory posits that an individual’s confidence level about overcoming challenges varies and can change depending on the input source. Levels of self-efficacy positively correlate with the likelihood of performing a behavior (Bandura, 1982). Extending Bandura’s construct, parental self-efficacy is defined as the extent to which parents believe their actions aid their children (Jones and Prinz, 2005). Parental self-efficacy is a vast concept profoundly altered depending on the situation, task, and context (Buchanan et al., 2024). For example, parents might feel highly confident managing their children’s morning routines but struggle to determine age-appropriate terminology when discussing sensitive topics such as suicide.

“Suicide-related prevention communication” is “an overarching, general term to describe all forms of conveying information to another person about the prevention, intervention, and postvention of suicidal ideation or behavior” (Frey et al., 2020, p. 813). Parents’ suicide-related prevention communication self-efficacy is characterized by their belief (or lack thereof) that they can partake in suicide-related prevention communication with their children (Czyz et al., 2018). Suicide-related prevention communication tasks entail discussion between two or more people about suicidal ideation, intent, and behaviors (Frey et al., 2020). The discussion may address one or multiple topics related to suicide, including personal experience, exposure, and/or prevention (Frey et al., 2020). Study findings suggest that parental participation in these types of communications with their teenagers improves young people’s behavioral health and overall well-being (Czyz et al., 2018).

As Czyz et al. (2018) have shown, the probability of parents participating in suicide-related prevention communication tasks can be determined by their levels of agreement or disagreement with statements such as “I can ask my teen if she or he is thinking about suicide.” High probability, which is associated with higher self-efficacy, indicates a strong likelihood that parents will introduce adolescents to suicide-related prevention communication and maintain essential discussion over time, even after a suicide attempt (Czyz et al., 2018). If the likelihood is low, parental suicide-related prevention communication self-efficacy can be strengthened through interventions such as parent education programs and family therapy (Czyz et al., 2018). Whether parents develop high suicide-related prevention communication self-efficacy independently or with professional assistance, they feel more equipped to assist adolescents in securing lethal means, adhering to safety plans, and receiving emotional support (Czyz et al., 2018). Thus, understanding and increasing parents’ suicide-related prevention communication self-efficacy can aid efforts aimed at reducing suicide risk among youth and highlight areas for targeted interventions to support parents in managing their teens’ suicidal crises.

### Measuring parental suicide prevention communication

As part of a more extensive study exploring how parents perceived their ability to support their teens during suicidal crises and how these perceptions relate to future emergency department visits and suicide attempts, Czyz et al. (2018) created a Parent Self-Efficacy Scale for suicide prevention activities (hereafter referred to as Parental Suicide Prevention Communication Self-Efficacy Scale [PSPCSE]). Their scale consists of nine items aimed at measuring parents’ confidence in engaging in specific suicide-related prevention tasks. The PSPCSE items were based on an extensive review of the existing literature on parental self-efficacy and suicide prevention to identify relevant constructs. Based on review findings and in consultation with clinicians and suicide risk management experts, the PSPCSE items were generated to cover various aspects of parental self-efficacy, such as recognizing warning signs, engaging in preventive actions, and maintaining safety (Czyz et al., 2018). Experts reviewed the item pool to refine the items and ensure clarity and comprehensibility.

In a sample of 162 parents, the PSCPSE demonstrated a strong Cronbach alpha of 0.87 and interitem correlations ranging from 0.29 to 0.77 (Czyz et al., 2018). The researchers did not complete a full validation of the instrument, and therefore, no additional psychometric properties were reported. While the tool is a step toward gaining a better understanding of how to support parents whose adolescent may be at risk for suicide, it was administered among a predominantly non-Latina Caucasian sample of mothers. Specifically, 2.5% of the sample identified as Latino; most of the parents were mothers (79.6%), with a few fathers (16.7%). Thus, this current study aims to slightly adapt the scale for use with Latino parents of adolescents living in the U.S. and to complete a full validation study to assess whether the scales would be suitable for use among this population.

#### Preventing suicide among Latino adolescents

Suicide among Latino youth in the U.S. is becoming a public health crisis. Since 2007, Latino youth suicidal behaviors in the U.S. have continued to rise (Brewer et al., 2022; Gomez et al., 2011). Consequently, the involvement of Latino parents in the prevention and treatment of these behaviors has become increasingly critical as they ‘often function as the “first and last resort”’ for youth (cited in Grant et al., 2015, p. 295). Latino families typically adhere to values of collective and family well-being (Allison and Bencomo, 2015; Buchanan et al., 2024). Therefore, involving family members, particularly parents, in preventing suicide among their adolescent children is crucial. Research findings continually reiterate the positive impact that family involvement has on reducing Latino youth suicide risk (see Villarreal-Otálora et al., 2023b). Findings from Danzo et al. (2024) suggest that parental proactive involvement significantly reduces suicidal behaviors among adolescent children. Conversely, negative attitudes or lack of engagement by caregivers were linked to higher risks of suicidality. Moreover, parents’ confidence in participating in suicide-related prevention communication impacts the adolescents’ treatment as parents are often tasked with carrying out specific suicide prevention behaviors, including monitoring their youth’s mood and behavior, securing lethal means in the home, and asking their youth directly about suicidal thoughts.

#### Latino parental involvement in suicide prevention: lacking specificity and ethnic focus

Despite acknowledging the importance of involving Latino parents in the prevention and treatment of suicide for their adolescent children, there continues to be a gap in knowledge regarding Latino parents’ needs and comfort with this topic. This gap is particularly evident in a lack of research that considers the multilingual and multinational diversity within the Latino population and how these factors may influence their involvement in suicide prevention with their children. There are 63.6 million Latinos in the U.S., representing a diverse array of immigration statuses and countries of origin (Krogstad and Noe-Bustamante, 2023). These individuals come from a variety of backgrounds, with significant populations originating from Mexico, Puerto Rico, Cuba, El Salvador, and the Dominican Republic, among others, reflecting the rich cultural and linguistic diversity within the Latino community in the U.S. (Krogstad and Noe-Bustamante, 2023; Moslimani and Noe-Bustamente, 2023). Nearly 70% of the U.S. Latino population primarily speaks Spanish, with a much lower percentage among U.S.-born Latinos (55%) compared to foreign-born Latinos (94%; Krogstad and Noe-Bustamante, 2023). This diversity within the Latino community underscores the need for culturally and linguistically tailored approaches to parental involvement in adolescent suicide prevention, as the varying backgrounds and language proficiencies may significantly impact their parents’ self-efficacy and comfort in addressing their adolescents’ mental health needs.

Research suggests that a parent’s self-efficacy is influenced by culture, language, child’s age, and in response to the youth’s mental health status (Boruszak-Kiziukiewicz and Kmita, 2020; Buchanan et al., 2024; Wittkowski et al., 2017). For example, recent study findings highlight that the parental self-efficacy levels of non-Latino parents in the U.S. are negatively correlated to their child’s mental health status (Boruszak-Kiziukiewicz and Kmita, 2020). In contrast, parental self-efficacy levels among Latino parent samples seem to remain the same regardless of their child’s mental health status (Villarreal-Otálora et al., 2023a; Boruszak-Kiziukiewicz and Kmita, 2020).

Among Latino parents, familism—a cultural value emphasizing the importance of the family unit—and parents’ levels of U.S. acculturation have been shown to strongly influence their parental self-efficacy levels (Boruszak-Kiziukiewicz and Kmita, 2020; Valdivieso-Mora et al., 2016). Specifically, in U.S.-based samples, researchers have found a negative correlation between Latino parents’ acculturation levels and parental self-efficacy levels; as the former increases, the latter decreases (Boruszak-Kiziukiewicz and Kmita, 2020); and a positive correlation between familism and parental self-efficacy related to suicide prevention (Villarreal-Otálora et al., 2023a).

These findings underscore the need to consider cultural nuances when working with and supporting Latino parents whose adolescents are at risk for suicidal behaviors. Considering these nuances can lead to more effective engagement and support strategies tailored to the unique experiences of Latino families.

### Current study

To gain a deeper and more practical understanding of parental self-efficacy for prevention interventions, scholars recommend task-related parental self-efficacy measures that are developmental-age specific over general ones (Wittkowski et al., 2017). However, findings from recent systematic reviews demonstrate a lack of studies examining parental self-efficacy among immigrant parents (see Boruszak-Kiziukiewicz and Kmita, 2020) and, more broadly, parents of adolescents (see Glatz et al., 2024); further, there is a need for more specific measures for parents of adolescents (Wittkowski et al., 2017). No validated task-specific measurement tool yet exists to assess Latino parents’ suicide-related prevention communication self-efficacy with their adolescent children. Thus, to create these necessary tools, the current study’s authors set out to validate a pair of scales slightly adapted from the PSPCSE scale (Czyz et al., 2018) for use with Latino parents of adolescent children in the U.S. The scales, collectively known as PSPCSE-Lat, consist of both English- (PSPCSE-Lat-E) and Spanish- (PSPCSE-Lat-S) language measures.

## Materials and methods

This cross-sectional survey study design was approved through the first author’s Institutional Review Board (IRB-FY23-69). The study was conducted through a comprehensive three-phase process designed to thoroughly investigate the complexities and diversity of Latino parental self-efficacy with suicide prevention communication. In the first phase, the original PSPCSE scale was reviewed and adapted in consultation with experts and gatekeepers in Latino family dynamics and mental health. In the second phase, the adapted scales were embedded into a larger survey administered to a sample of Latino parents of adolescent children living in the U.S. In the third phase, differences in self-efficacy between foreign-born and U.S.-born Latino parents regarding specific suicide prevention communication tasks were explored.

Eligibility screening, informed consent, and data collection were done via the Qualtrics survey platform. The first part of the survey asked several eligibility screening questions. If participants met the study inclusion criteria, they were directed to the informed consent information provided in two formats: written and audio-visual. Possible participants were informed of the potential psychological discomfort that might arise from reading suicide-related survey questions, and, in response to this risk, regardless of whether an individual opted to participate in the study, they were provided with a list of national suicide-prevention resources (i.e., hotlines and websites). Additionally, participants were informed about the estimated time (approximately 20 min) required to complete the survey. If, after reviewing the informed consent form, they agreed to participate in the study, they were prompted to click next to begin answering the questionnaire.

### Participants and procedures

Participant recruitment occurred via Qualtrics panels and through social media posts and emails. Recruitment efforts occurred between November 2022 and March 2023. Interested participants were asked to click on the survey link. On the survey landing page, potential participants answered a set of screening questions, and if they met the eligibility criteria, they were directed to the informed consent page. After consent, they could self-select to participate in the study and begin completing the study questionnaire. To be eligible for the study, individuals had to: (a) be at least 18 years old; (b) live in the U.S.; (c) self-identify as Hispanic/Latino; (d) be a parent (i.e., biological parent, legal guardian, or caregiver who has childrearing responsibilities) of a child between the ages of 10 and 18; and meet one of the following combinations: (i) be ‘‘U.S.-born,” meaning born in the U.S. (excluding Puerto Rico) and read/speak English; *or* (ii) be “foreign-born,” defined, for the purposes of this study, as born in the Spanish or Portuguese speaking countries of Mexico, the Caribbean, South and Central America, or born on the culturally- and linguistically-Latino U.S. territory of Puerto Rico. Participants were assigned to the English or Spanish version of the survey based on a combination of birthplace and the binary language screener questions. Specifically, U.S.-born participants who answered “Yes” to the question “Can you read and speak English?” were assigned the English version of the survey. Foreign-born participants from Spanish- or Portuguese-speaking countries (or Puerto Rico) who answered “Yes” to the question “¿Usted puede leer y escribir en Español?” were assigned the Spanish version. Language fluency was not formally assessed beyond these screener questions; however, participants who responded negatively were excluded from the corresponding survey version to ensure comprehension and data quality. To protect psychological well-being, parents who had a child die by suicide were excluded from the study. Participants were offered a $5 e-gift card for completing the study survey as a recruitment incentive.

A total of 634 Latino parents of adolescent children living in the U.S. completed the survey. Foreign-born (*n* = 220) participants completed the Spanish version of the survey; U.S.-born (*n* = 414) participants completed the English version of the survey. Participants’ demographic information is presented in Table 1.

**Table 1:** Participants’ demographic characteristics (*N* = 634).

Characteristic	English	Spanish
Age		
*M* (SD)	39.76 (6.70)	40.44 (8.41)
Range	23–69	22–71
Sex		
Female	175 (43.1%)	128 (58.2%)
Male	230 (56.7%)	91 (41.4%)
Education		
Middle school or lower	3 (0.7%)	55 (26%)
High school	55 (13.6%)	55 (26%)
Post-secondary education	347 (85.5%)	101 (47.7%)
Marital status		
Single, never married	39 (9.6%)	22 (10.4%)
In a relationship	23 (5.7%)	39 (18.4%)
Married	312 (77.1%)	129 (60.8%)
Separated or divorced	28 (6.9%)	20 (9.4%)
Widowed or other	3 (0.7%)	2 (0.9%)
Religion		
Catholic	167 (41.1%)	130 (59.1%)
Protestant	24 (5.9%)	28 (12.7%)
Orthodox	23 (5.7%)	0 (0.0%)
Jehovah’s witness	9 (2.2%)	7 (3.2%)
Atheist	80 (19.7%)	5 (2.3%)
None	74 (18.2%)	26 (11.8%)
Other	29 (7.1%)	24 (10.9%)

English responses were 414. Spanish responses were 220.

### The measurement tool and survey instrument

As part of a study exploring parental perceptions of their ability to support teens during suicidal crises and the relation to future emergency department visits and suicide attempts, Czyz et al. (2018) developed the PSPCSE. This 9-item, 11-point Likert scale measures parents’ confidence in engaging in specific suicide-related prevention tasks based on an extensive literature review and consultations with clinicians and suicide risk management experts. The PSPCSE was validated in the U.S. in a sample of 162 parents. Reliability of the original scale was strong, as indicated by a Cronbach alpha of 0.87 and interitem correlations ranging from 0.29 to 0.77. Although the tool was not developed for a validation study, it provides insights into supporting parents of adolescents at risk for suicide.

Though their work was groundbreaking, the Czyz et al. (2018) study sample included only 2.5% Latinas, predominantly mothers (79.6%), and was conducted exclusively in English. This raises the question of whether the tool is applicable and effective for Latino parents. In 2020, with permission from the originator, the primary author made slight adjustments in wording on the PSPCSE to be age-specific (swapping out the word “child” for “adolescent”) and to include lethal-means safety items. The adapted instrument was then translated using a conceptual and back translation approach to create revised versions—in both English and Spanish—of the PSPCSE that were designed to examine Latino immigrant parents’ engagement, knowledge, and confidence in Latino adolescent suicide prevention for a study (Villarreal-Otálora et al., 2023a). For the current study, the research team finetuned these new English (PSPCSE-Lat-E) and Spanish (PSPCSE-Lat-S) versions of the scales and validated them for use with Latino parents living and caring for an adolescent child in the U.S.

To finetune the scales, we shared the scales with two bilingual (English and Spanish) mental health experts who specialize in suicide prevention with Latino families. They reviewed the items for Latino cultural sensitivity, linguistic accuracy, and relevance on both scales. For example, experts were asked if the scale instructions and the items addressed relevant cultural practices, such as respect and personalism. Based on their recommendations, one of the items in Spanish was reworded to soften the statement for Latino parents. Initially, the item in English read, “Prevent your adolescent from accessing lethal means if they have suicidal thoughts in the future.” The experts perceived this original wording too confrontational and stigma-invoking for the intended population, given that the item included several stress-invoking terms all in one: “prevent, lethal means, and suicidal thoughts.” Thus, the item was changed to “If your adolescent is thinking about suicide, keep them from gaining access to lethal means (e.g., knives, guns).” These changes softened the tone and provided an example of a term, lethal means, uncommon among this population. Similarly, the term ‘self-efficacy’ was changed to ‘confidence’ in the title of the scale because experts found the original term too complex and unrelatable.

Furthermore, experts suggested using the non-literal translation ‘*seguro*’ (secure) instead of ‘confident’ for the main prompting question of the Spanish version. This change was made to enhance clarity and specificity for the Latino culture. In Spanish, ‘*seguro*’ is more specific to the context of self-efficacy, conveying a sense of assurance. In contrast, ‘*confianza*’ (confidence) is a broader term that includes trust in others and does not solely focus on personal ability. Lastly, one item was rewritten to adhere to the formal ‘*usted*’ tense in Spanish, ensuring cultural sensitivity to the value of respect. After rewording the statements in the Spanish version, we conceptually translated the items back to English and reversed them back to Spanish to verify the accuracy of the translation (Knight et al., 2009).

Next, we focused on ensuring the face validity of the scales with ten Latino parents (five for the English version and five for the Spanish version). These parents self-selected to participate in the pilot after seeing a flyer regarding the study. The pilot participants were asked to complete the PSPCSE-Lat and then asked: (1) to rate each item and the response choices on clarity, understanding, and appropriateness; (2) provide their thoughts on the survey flow and item sequencing; and (3), offer their overall impressions of the scale. After this process, the 11-items were finalized in both the PSPCSE-Lat-E and PSPCSE-Lat-S scales, and we proceeded to create the final survey and conduct the validation study.

The survey consisted of 60 questions, including:

1  PSPCSE-Lat-E (11 items) or PSPCSE-Lat-S (11 items).
2  Questions regarding the participant’s children’s ages, genders, and suicidal behaviors (9 items).
3  Scales for construct validation:

  ◦  12-Item Brief Literacy of Suicide Scale (BLOSS): This scale assesses knowledge and understanding of suicide, covering four domains: signs/symptoms, causes/nature, risk factors, and treatment/prevention (Batterham et al., 2013).
  ◦  5-Item Pan-Hispanic Familism Scale: This scale measures attitudes about the importance of family in Hispanic populations, focusing on familial obligations, support from the family, and family as referents (Villarreal et al., 2005).

4  Questions related to suicide awareness and preferences for suicide prevention programming. (11 items).
5  Sociodemographic questions. (12 items).

### Analytical plan

While the primary aim of this study was to uncover the factor structure of the newly created PSPCSE-Lat scales, we first reviewed descriptive characteristics of our samples regarding interests in suicide prevention programs, program preferences, and questions related to mental health and suicide.

We then implemented an Exploratory Factor Analysis (EFA) to uncover the underlying factor structures within each survey version. Initially, we conducted separate preliminary data screenings for each sample to check for missing values and outliers and to ensure that the data met the assumptions necessary for EFA, such as normality and linearity. We assessed the suitability of the datasets for factor analysis by calculating the Kaiser-Meyer-Olkin (KMO) measure of sampling adequacy along with Bartlett’s test of sphericity for both the English and Spanish samples. Traditional KMO metrics (KMO > 0.80) were considered for sample adequacy.

EFAs were then performed separately on each language sample using maximum likelihood as the extraction method. As the survey was designed to capture one unique construct, rotation was not initially employed. The number of factors to retain was determined using a combination of Kaiser’s criterion (eigenvalues >1), examination of the scree plot for inflection points, and parallel analysis to compare the eigenvalues with those obtained from randomly generated data. Only loadings of 0.40 or higher on a factor we considered significant (Howard, 2016). We also looked for cross-loadings, where items loaded significantly on more than one factor, and made decisions about item retention based on theoretical consistency and the aim to achieve a simple structure. After identifying the factor structures within each language group, we compared the factors across the English and Spanish versions to evaluate their similarity.

To assess the equivalence of the factor structures between the two language groups, we calculated Tucker’s coefficient of congruence for each pair of corresponding factors. Coefficients close to 1 indicated a high degree of similarity across groups. Additionally, we computed McDonald’s *ω* coefficients for each identified factor in both samples to evaluate internal consistency, considering values above 0.70 as adequate (Kalkbrenner, 2021).

Lastly, to evaluate measurement equivalence between U.S.- and foreign-born participants, independent samples *t*-tests and Item Response Theory (IRT) analysis were conducted to compare item-level responses between language groups. For each individual item in the scale, a *t*-test was performed to examine whether there were statistically significant differences in mean responses between participants who completed the English version versus those who completed the Spanish version. To account for multiple testing, we consider items significant based on a Bonferroni correction, *p* < 0.0045. We employed IRT modeling to psychometrically evaluate latent traits by analyzing participants’ response patterns to test items to account for both item characteristics and individual differences in item performance.

## Results

A total of 634 Latino parents living in the U.S. completed the survey. Foreign-born (*n* = 220) participants completed the Spanish version of the survey; U.S.-born (*n* = 414) participants completed the English version of the survey (see Table 1 for sample details). Participants were evenly split between males (51.32%) and females (48.41), with approximately 0.27% indicating other. Age ranged between 22–71 years old (*M* = 40.00, SD = 7.36). Most participants were born in the U.S. (64.03%) or Mexico (14.51%), and most reported living in California (18.45%), Texas (14.83%), or Florida (11.04%). Nearly half of the sample identified as Catholic (46.85%). Most participants reported being married or cohabitating with a partner (66.88%) and making less than $85,000 per year in household income (63.75%). More than one-third of the sample reported completing college (36.74%).

### Preliminary analysis

An EFA was conducted to evaluate underlying latent structures using an English and Spanish version of the PSPCSE-Lat. Data were initially screened for missing values, outliers, and assumptions. There were no missing values for key items used in subsequent analyses. Items were normally distributed with skewness and kurtosis values falling within acceptable ranges (±2) (Kline, 2023). The Kaiser-Meyer-Olkin (KMO) measure verified sampling adequacy for the English (Overall MSA = 0.90) and Spanish (Overall MSA = 0.83) samples. Bartlett’s test of sphericity was significant for both groups (English: χ^2^ = 1888.07, *p* < 0.001; Spanish: χ^2^ = 792.29, *p* < 0.001), supporting the factorability of the correlation matrices.

### English version

The EFA for the PSPCSE-Lat-E yielded one factor with an eigenvalue of 5.226, accounting for 42.4% of the total variance. All items contributed significantly to the single underlying construct, with loadings ranging from 0.520 to 0.738. Moreover, the majority of variance in the items was explained by prominent factor uniqueness values ranging from 0.456 to 0.730.

Fit indices indicated good model fit with a RMSEA of 0.037 (90% CI, 0–0.063), SRMR of 0.016, a TLI of 0.90, BIC of −58.10, and a CFI of 0.94. Internal consistency was strong (*ω* = 0.88). These results suggest that the English version of the survey is primarily unidimensional, with one dominant factor explaining the majority of the variance in the items. See Table 2 for the finalized PSPCSE-Lat-E scale.

**Table 2:** Parental suicide prevention communication self-efficacy Latino English scale.

How confident do you feel to …?	Not at all confident				Somewhat confident				Completely confident		
1. Ask your adolescent about their mood	0	1	2	3	4	5	6	7	8	9	10
2. Ask your adolescent if they are experiencing thoughts of suicide	0	1	2	3	4	5	6	7	8	9	10
3. Respond in a helpful manner if your adolescent discloses thoughts of suicide	0	1	2	3	4	5	6	7	8	9	10
4. Identify suicide indicators or warning signs in your adolescent	0	1	2	3	4	5	6	7	8	9	10
5. Obtain a commitment from your adolescent not to attempt suicide	0	1	2	3	4	5	6	7	8	9	10
6. Work with your adolescent on a plan for their safety	0	1	2	3	4	5	6	7	8	9	10
7. Assist your adolescent in accessing treatment services for their difficulties	0	1	2	3	4	5	6	7	8	9	10
8. Encourage your adolescent to cope with their difficulties in ways that have been helpful in the past	0	1	2	3	4	5	6	7	8	9	10
9. Offer emotional support to your adolescent (such as listen to them, tell them they are important to you, give them a hug)	0	1	2	3	4	5	6	7	8	9	10
10. Put away all of the house knives, over-the-counter and prescribed medications, and firearms in a lockbox	0	1	2	3	4	5	6	7	8	9	10
11. If your adolescent is thinking about suicide, keep them from gaining access to lethal means (e.g., knives, guns)	0	1	2	3	4	5	6	7	8	9	10

Villarreal-Otálora et al. (2023a).

### Spanish version

The EFA for the Spanish version of the survey revealed a more complex structure compared to the English version. The analysis identified three factors with eigenvalues exceeding 1, accounting for 51.6% of the total variance. Specifically, the first factor explained 34.7% of the variance with an eigenvalue of 4.297, the second factor accounted for 8.9% with an eigenvalue of 1.464, and the third factor contributed 8.1% with an eigenvalue of 1.077.

Moreover, factor loadings demonstrated clear distinctions among the three factors. Factor 1 was defined by items such as 1 (0.435), 5 (0.602), 6 (0.535), 9 (0.644), 8 (0.634), and 7 (0.615), indicating a coherent construct. Factor 2 was predominantly shaped by items 10 (0.997) and 11 (0.499), while Factor 3 included items 2 (0.469), 3 (0.420), and 4 (0.500).

**Figure 1:**
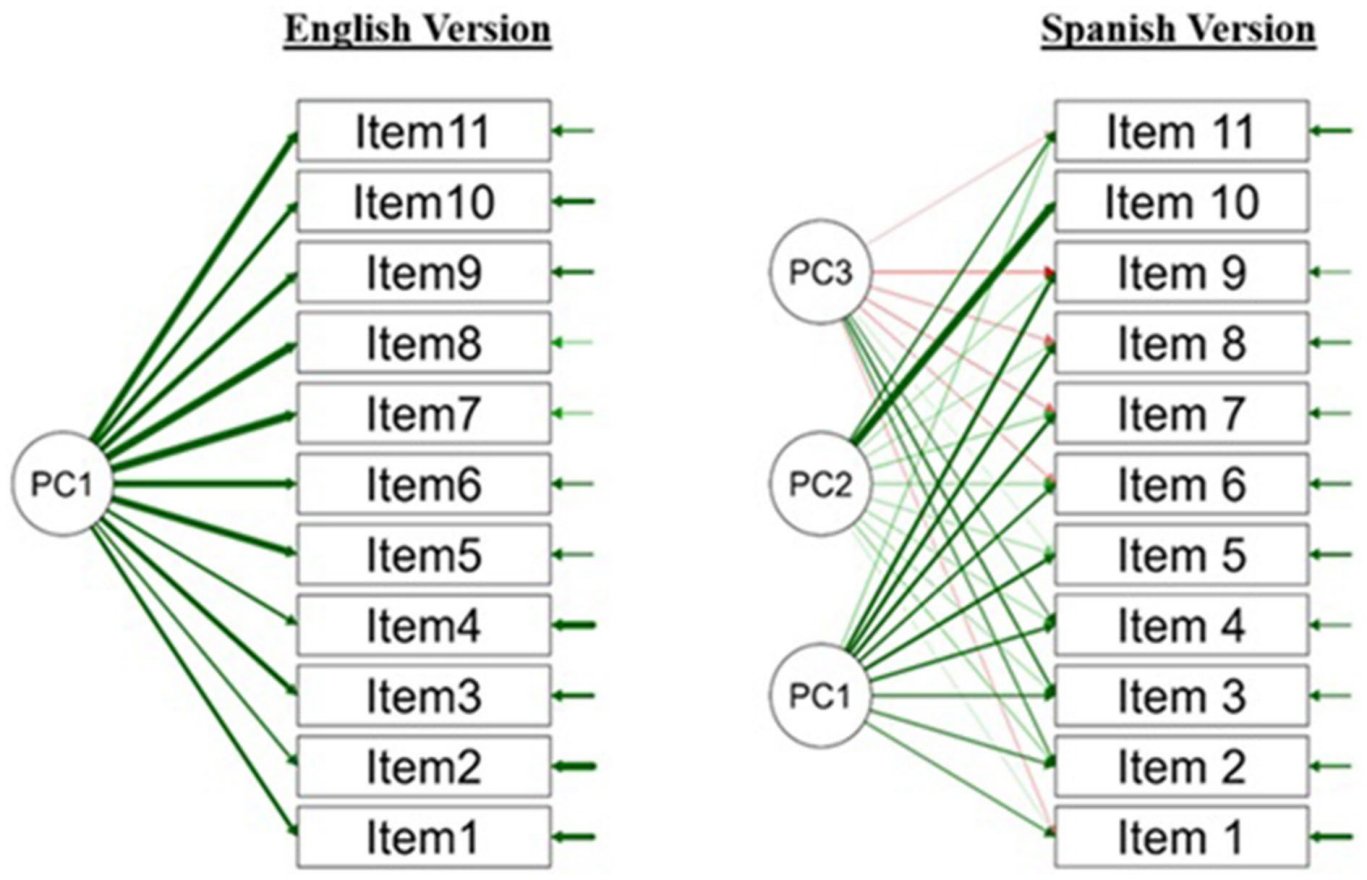
EFA models for English and Spanish versions of the PSPCSE-Lat.

Model fit indices supported the three-factor solution, with an RMSEA of 0.07 (90% CI, 0.04–0.09), SRMR of 0.03, TLI of 0.92, and CFI of 0.96, all indicating a good fit. Notably, the Bayesian Information Criterion (BIC) was −82.57. A negative BIC, although less common, signifies a strong model fit where the model’s likelihood greatly outweighs the penalty for complexity. This result implies that the Spanish version’s complex factor solution fits the data well. Again, the internal consistency was high (ω = 0.82). A path diagram for both model versions can be found in Figure 1.

In comparison, the English version displayed a simpler, unidimensional structure with a single dominant factor accounting for 42.4% of the variance. The divergence in factor structures suggests potential differences in how primary English and Spanish speakers interpret and respond to items related to talking more directly about suicide. Despite these differences, both versions demonstrated strong model fit, highlighting the survey’s overall robustness and suitability for use across different linguistic groups. This cross-language validity enhances the survey’s applicability in multicultural research settings, offering valuable insights into the underlying constructs across diverse populations. A path diagram for both survey versions can be found in Figure 1.

Tucker’s coefficients of congruence were calculated for one-, two-, and three-factor solutions. The one-factor solution unsurprisingly held the largest congruence (0.99), and the three-factor structure yielded the worst congruence (0.11–0.79). However, the two-factor structure yielded an interestingly high congruence as well, 0.97 and 0.94, respectively, with little overlapping congruence across factors. This prompted a follow-up analysis considering an orthogonal approach. See Table 3 for the finalized PSPCSE-Lat-S scale.

**Table 3:** Parental suicide prevention communication self-efficacy Latino Spanish scale.

¿Qué tan seguro se siente en …	Para nada seguro/a				Algo seguro/a				Completamente seguro/a		
1. Preguntarle a su adolescente de su estado de ánimo	0	1	2	3	4	5	6	7	8	9	10
2. Preguntarle a su adolescente si está experimentando pensamientos de suicidio	0	1	2	3	4	5	6	7	8	9	10
3. Responder de manera útil si su adolescente revela pensamientos de suicidio	0	1	2	3	4	5	6	7	8	9	10
4. Identificar señales de advertencia de suicidio en su adolescente	0	1	2	3	4	5	6	7	8	9	10
5. Obtener un compromiso de su adolescente a que no atente el suicidio	0	1	2	3	4	5	6	7	8	9	10
6. Trabajar con su adolescente en un plan para mantener su seguridad	0	1	2	3	4	5	6	7	8	9	10
7. Asistirle a su adolescente a conseguir tratamiento para sus dificultades	0	1	2	3	4	5	6	7	8	9	10
8. Animar a su adolescente a lidiar con sus dificultades de manera que haya sido útil en el pasado	0	1	2	3	4	5	6	7	8	9	10
9. Ofrecer apoyo emocional a su adolescente (como escucharlos, decirles que son importantes para usted, abrazarlos)	0	1	2	3	4	5	6	7	8	9	10
10. Guardar en una caja fuerte todos los cuchillos de la casa, medicamentos de vente libre, y armas (ej., pistola)	0	1	2	3	4	5	6	7	8	9	10
11. Si su adolescente tuviera pensamientos sobre el suicidio, mantener que no tenga acceso a medios letales (ej., cuchillos, pistolas)	0	1	2	3	4	5	6	7	8	9	10

### Follow-up analysis EFA

Because the congruence analyses indicated a clearly orthogonal relationship between two unique factors, we conducted a follow-up EFA. The factor structure was still estimated via maximum likelihood, but we included a varimax rotation to assess whether the hypothetical orthogonal relationship appeared in both samples without substantially impacting model fit.

**Figure 2:**
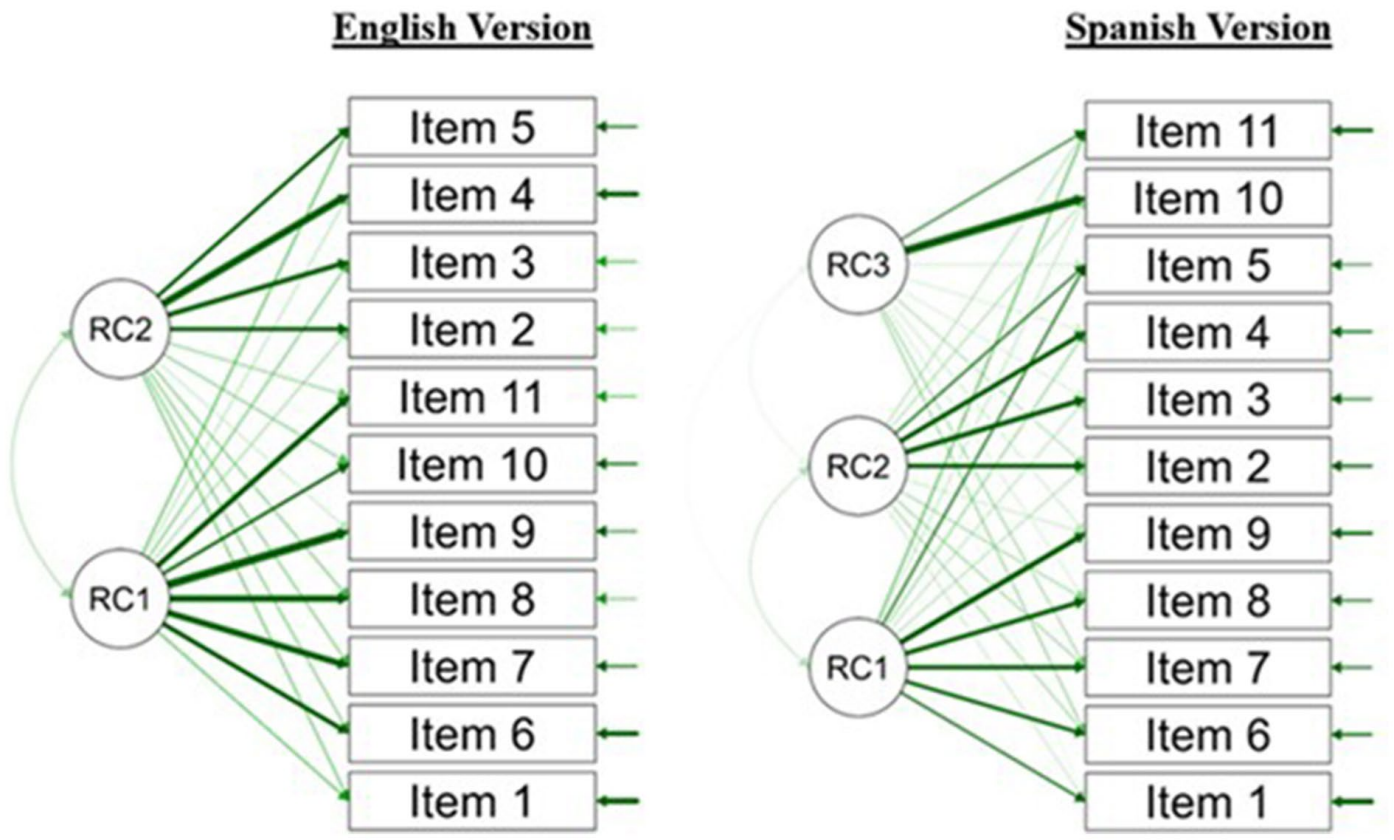
Rotated EFA models for English and Spanish versions of the PSPCSE-Lat.

While the model fit in the Spanish sample was unchanged, the English version was impacted, albeit it still maintained a suitable fit. Based on Eigenvalues, a two-factor structure was the best fit for the English version, with Factor 2 now explaining 5.9% of the proportion variance. Importantly, similar to Factor 3 in the Spanish version, Factor 2 now included the suicide-specific Items 2 (0.580), 3 (0.619), and 4 (0.720). Note that after the orthogonal rotation, the Spanish version maintained the three-factor solution (see Figure 2 for the path diagram).

### Measurement invariance across languages

Across language groups, the 3-factor Spanish model demonstrated a good configural fit (e.g., CFI = 0.93; RMSEA = 0.09), indicating that the same three-factor pattern holds for both the English and Spanish versions. Constraining factor loadings (metric invariance) did not degrade fit (CFI = 0.93; RMSEA = 0.08), supporting equality of loadings and thus comparable associations among constructs across languages. When intercepts were constrained (scalar invariance), fit declined (CFI = 0.91; ΔCFI = 0.018; RMSEA = 0.09), suggesting only partial scalar invariance at best. Strict invariance (equal residuals) was clearly unsupported (CFI = 0.79; RMSEA = 0.13), as was structural invariance (CFI = 0.67; RMSEA = 0.15). Taken together, the English and Spanish forms may be reasonable for testing relations (e.g., regressions, correlations) at the latent level. However, direct comparisons of latent means should only be done with proper partial-invariance adjustments or alignments (e.g., down-weighting noninvariant items) rather than assuming strict or structural equality (Asparouhov and Muthén, 2014).

Invariance was not supported for the unidimensional version established for the unrotated English version, which had poor configural fit (e.g., CFI = 0.84, RMSEA = 0.12), along with drastic decreases in metric, scalar, and structural fit. Similarly, the two-factor model evaluated with the follow-up EFA analysis did not produce a suitable invariance fit. Therefore, while a direct comparison can be made with the 3-factor solution, we suggest that researchers make appropriate adjustments before doing so.

### Item level group differences

Independent samples *t*-tests were conducted to examine differences in item responses between versions. The analysis revealed distinct patterns in version differences (see Table 4). These differences represented small to medium effect sizes, with Item 3, *Respond in a helpful manner if your adolescent discloses thoughts of suicide*, showing the largest effect. Notably, these items also represent the items identified in the rotated factor structures in both groups for items that directly mention the word “suicide,” suggesting English version respondents may be more comfortable discussing these issues with their children (Items 2, 3, and 4).

**Table 4:** Item differences between foreign-born and U.S.-born Latino PSPCSE.

Item	Welch’s *t*	df	*p*	Cohen’s *d*	English		Spanish	
					*M*	*SD*	*M*	*SD*
*1	−3.05	381.11	0.002	−0.26	7.76	1.83	8.29	2.2
*2	3.79	352.23	<0.001	0.33	6.96	2.71	5.91	3.61
*3	5.32	294.13	<0.001	0.48	7.7	2.2	6.18	3.92
*4	4.16	304.59	<0.001	0.37	7.32	2.08	6.26	3.47
5	0.12	353.32	0.908	0.01	7.53	2.25	7.50	3.00
6	−0.51	354.02	0.612	−0.04	8.19	1.84	8.29	2.45
*7	−2.97	369.54	0.003	−0.26	7.98	1.86	8.52	2.33
*8	−3.61	403.81	<0.001	−0.31	7.95	1.84	8.55	2.07
*9	−6.57	488.26	<0.001	−0.54	8.38	1.71	9.26	1.54
10	1.11	310.57	0.270	0.10	8.05	1.93	7.79	3.12
11	−1.76	340.79	0.079	−0.15	8.25	1.92	8.61	2.69

*Indicates significance at a Bonferroni corrected *p* < 0.0045. *N* = 634. 414 English participants and 220 Spanish participants.

Conversely, foreign-born participants scored significantly higher on several items, as indicated by negative t-values. The largest difference was observed for Item 9, *Offer emotional support to your adolescent (such as listen to them, tell them they are important to you, give them a hug),* showing a medium effect size. No significant differences were found for Items 5, 6, 10, and 11. It should be noted that several items (1, 2, 3, 4, and 9) violated the assumption of equal variances, and violations of normality were detected for all items. Thus, all items were compared with Welch’s *t*-test.

### Item response theory

**Figure 3:**
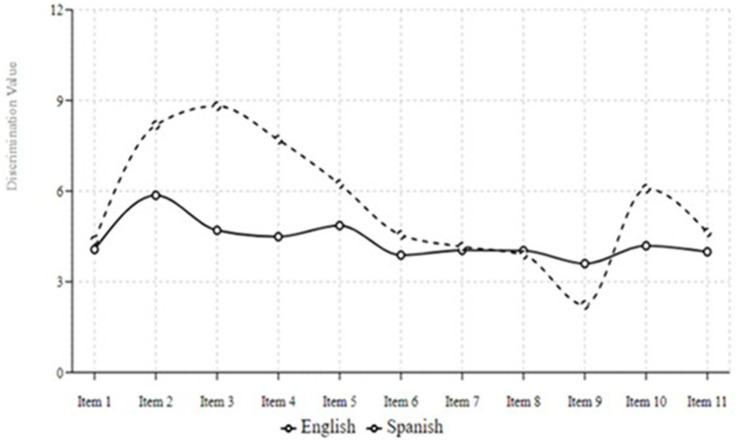
Item discrimination values by question and language version.

Last, to further examine the psychometric properties of the PSPCSE-Lat versions, we conducted an Item Response Theory (IRT) analysis. Item discrimination values (see Figure 3) provide insight into the consistency in response patterns parents had on each item in the survey. The Spanish version showed substantially higher discrimination values for Items 2–4 (values ranging from 7.70 to 8.81) compared to the English version (values ranging from 4.49 to 5.86). This indicates these items are particularly effective at distinguishing between Spanish version parents with high versus low confidence in suicide-specific communication despite their lower mean scores on these items. This may suggest that the variability in responses that participants in the Spanish version we detected is due to a lack of comfort with those items that directly mention the word “suicide.” Interestingly, Item 9 showed the opposite pattern, with lower discrimination in the Spanish version (2.23) than in the English version (3.60), despite higher mean scores among Spanish-speaking respondents. This suggests a potential ceiling effect in the Spanish version of this item, where most respondents reported high confidence regardless of their overall communication comfort level.

## Discussion

This study aimed to validate the PSPCSE-Lat-E and PSPCSE-Lat-S scales. Our results support the validity of the English and Spanish versions of the 11-item PSPCSE-Lat scales for use with Latino parents of adolescents. Interestingly, the findings also reveal nuances in how foreign-born, primarily Spanish-language Latino parents conceptualize suicide prevention tasks as compared to those who are U.S.-born, primarily English-language Latino parents. These differences are evident in the dimensional structure of the PSPCSE-Lat-S scale, which has three separate but related dimensions, versus the English version’s two-dimensional structure. These variances between foreign- and U.S.-born Latino parents provide significant implications for Latino families, for their health providers, for suicide prevention advocates, and for researchers. The scales can now be used in research, clinical, and community settings to improve our understanding of Latino parents’ suicide-related prevention communication self-efficacy.

### Factor structure and cultural implications of PSPCSE-Lat versions

The three-factor structure of the PSPCSE-Lat-S is as follows: (1) Family-orientated communication and support; (2) Lethal means restriction; and (3) Direct awareness and intervention. Notably, only two of these three dimensions are present in the PSPCSE-Lat-E scale: (1) Lethal means restriction and (2) Direct awareness and intervention.

*Family-orientated communication and support,* is unique to the PSPCSE-Lat-S, and the suicide-related prevention communication tasks (i.e., Items 1 and 5–9) seem closely aligned with the attitudinal value of familism. These tasks measured by this factor focus on the parents’ emotional and instrumental social support to their adolescents during difficult situations (Valdivieso-Mora et al., 2016). In the context of familism, open communication among family members is emphasized to prioritize and understand emotional well-being within the cultural framework of the family. This cultural value is reflected in the practice of asking adolescents about their mood (Item 1) and ensuring that their emotional state is acknowledged and supported within the family unit. Similarly, Item 8, *Encourage your adolescent to cope with their difficulties in ways that have been helpful in the past*, emphasizes a family-centered approach to problem-solving, drawing on past experiences within the family to bolster the adolescent’s emotional resilience. Even Item 5, which explicitly mentions “suicide,” underscores the family’s protective function embedded in familism. It emphasizes the importance of prioritizing the adolescent’s safety and mental health by fostering a trusting environment and ensuring the adolescent feels supported within the family unit.

The fact that *family-orientated communication and support* only manifested in the factor structure of the PSPCSE-Lat-S scale is consistent with other research (Allison and Bencomo, 2015) that shows higher rates of familism among foreign-born Latino parents versus those who were born in the U.S. There is a positive correlation between internalizing mental health concerns and familism for Latino youth (Villarreal-Otálora et al., 2023b; Valdivieso-Mora et al., 2016). Therefore, directly measuring these protective tasks among a Latino parent of youth at risk for suicide may be helpful in that it creates space to explore the possibility of working toward increasing behavioral and attitudinal familism as steps the parent may take to engage in suicide prevention.

*Lethal means restriction* (PSPCSE-Lat-S Items 10 and 11; PSPCSE-Lat-E Items 10 and 11) measures confidence in carrying out direct behaviors to limit youth access to objects that could be used for suicide. Given that lethal means counseling is often neglected by routine care but is a key component in preventing deaths by suicide (Asarnow et al., 2021; Hunter et al., 2021), it is crucial to gauge the parents’ confidence in performing this task. It is essential to recognize that lethal means restriction is a critical intervention strategy in suicide prevention. By limiting access to potentially harmful objects, parents can significantly reduce the risk of a suicide attempt and death. Parents’ confidence in implementing these restrictions is paramount, as it directly influences the effectiveness of the intervention. Thus, depending on the parents’ confidence level, they could be referred to psychoeducational programs to help them determine when and how to engage in this life-saving prevention task.

Psychoeducational programs can provide valuable resources and training. These programs can help parents understand the importance of lethal means restriction, identify potential hazards in the home, and develop strategies to secure these items effectively. Additionally, these programs can offer guidance on communicating with their adolescents about the reasons for these safety measures, fostering an environment of trust and understanding. The “Lock and Protect” digital decision aid (DA) is an excellent example of a resource designed to assist parents in this critical task (Asarnow et al., 2021). This web-based tool is available in both Spanish and English and was developed with input from Latino stakeholders, making it accessible to a broader audience (Asarnow et al., 2021). The DA provides step-by-step instructions on how to conduct lethal means counseling (Asarnow et al., 2021), helping parents feel more confident and capable in their role as protectors of their children’s mental health. By integrating such tools and programs into routine care, healthcare providers can ensure that Latino parents are well-equipped to perform lethal means restriction, ultimately contributing to the reduction of suicide rates among adolescents. Regular follow-up support and check-ins can further reinforce the importance of these safety measures and address any challenges parents may face.

*Direct awareness and intervention* (PSPCSE-Lat-S Items 2–4; PSPCSE-Lat-E Items 1—9) measures the confidence level to engage in direct suicide prevention tasks. An essential prevention skill is to identify suicide warning signs and ask directly about the presence of suicidal ideation (Frey et al., 2020). This skill is crucial because most youth report that they often will not provide a direct suicide communication disclosure unless they are directly probed about the topic (Ballard et al., 2013). Moreover, most reports want to be asked directly without judgment as this will increase the likelihood of a suicide risk identification and an opportunity to connect with help (Ballard et al., 2013; O’Reilly et al., 2016). Interestingly, foreign-born parents struggled more than their U.S.-born counterparts in answering these questions.

### Effectiveness of suicide-specific communication items: cultural and linguistic specificity

The findings from our IRT analysis provide valuable insights into the psychometric properties of the PSPCSE-Lat versions. The analysis revealed notable differences in item discrimination values between the Spanish and English versions of the scale, highlighting the effectiveness of specific items in distinguishing between parents with varying levels of confidence in suicide-specific communication.

Items 2–4 in the Spanish version demonstrated substantially higher discrimination values (ranging from 7.70 to 8.81) compared to the English version (ranging from 4.49 to 5.86). This finding suggests that these items are particularly effective at differentiating between Spanish-speaking parents with high versus low confidence in discussing suicide. Despite the lower mean scores on these items, the high discrimination values indicate that the variability in responses may be attributed to a lack of comfort with directly mentioning the word “suicide.” This finding underscores the importance of cultural and linguistic considerations when developing and validating scales for diverse populations.

Conversely, Item 9, *Offer emotional support to your adolescent (such as listen to them, tell them they are important to you, give them a hug),* exhibited lower discrimination in the Spanish version (2.23) compared to the English version (3.60) despite higher mean scores among Spanish-speaking respondents. This pattern suggests a potential ceiling effect in the Spanish version of this item, where most respondents reported high confidence regardless of their overall comfort level with suicide-specific communication. The ceiling effect may limit the item’s ability to distinguish between different confidence levels, indicating the need for further refinement or alternative phrasing to enhance its discriminatory power.

Overall, these findings highlight the importance of conducting thorough psychometric evaluations across different language versions of a scale. The observed differences in item discrimination values emphasize the need to consider cultural and linguistic factors that may influence response patterns. Future research should explore strategies to address these disparities, such as modifying item content or providing additional context to improve comfort and understanding among respondents.

By refining the PSPCSE-Lat versions, we can enhance their utility in assessing parental self-efficacy in suicide-specific communication across diverse populations. This refinement, in turn, can inform targeted interventions and support efforts to improve suicide literacy and prevention strategies within different cultural contexts.

### Direct versus indirect suicide prevention tasks

Expanding on previous research findings (Villarreal-Otálora et al., 2023a, 2024; Boruszak-Kiziukiewicz and Kmita, 2020; Wittkowski et al., 2017), we not only see a difference in Latino parents’ self-efficacy along specific suicide prevention tasks but also their overall comfort with the word “suicide.” Supporting the notion that individual and community-level factors influence self-efficacy, foreign-born Latino parents whose primary language is Spanish seem to feel most comfortable engaging in passive-family orientated suicide prevention tasks. This finding is consistent with previous studies, which highlight that Latino parents are more likely to participate in community-based mental health workshops that focus on general well-being and family support rather than those explicitly addressing suicide (Villarreal-Otálora et al., 2024). Such workshops build on Latino parents’ passive-suicide prevention skills and slowly introduce direct-suicide prevention tasks in a culturally sensitive environment where they could learn about mental health in a way that aligns with their values and comfort levels.

Foreign-born Latino parents seem to feel least comfortable with direct suicide prevention tasks as compared to U.S.-born counterparts, as evidenced by their struggles with Items 2 through 5, which include “suicide”-direct prevention tasks. Preliminary research findings from previous studies (Villarreal-Otálora et al., 2024) suggest that immigrant Latina mothers experience embodied distress when the word “suicide” is mentioned. This distress can manifest physically and emotionally, making it challenging for them to engage in conversations or interventions related to suicide prevention. The cultural stigma surrounding mental health and suicide within many Latino communities exacerbates this distress, as discussing such topics is often considered taboo (Brewer et al., 2022). Thus, when working with Latino parents, especially foreign-born ones, it is best to use non-directive wording when bringing up the subject of suicide (e.g., instead of “suicide,” say “well-being”) and spend time assessing their emotional regulation.

Another explanation for the lack of comfort with direct suicide prevention tasks may be less exposure to indirect learning (observing others engage in the behavior). Foreign-born Latino parents may have less exposure to indirect learning opportunities, such as observing others engage in suicide prevention behaviors. Indirect learning is a key behavioral learning mechanism, especially for individuals with strong collectivistic viewpoints, like many foreign-born Latino parents (Bandura, 1982; Klassen, 2004; Nielsen Diverse Intelligence Series, 2021). In collectivistic cultures, learning often occurs through community interactions and shared experiences. However, due to language barriers, cultural differences, and limited access to mental health resources, these parents may not have had the opportunity to observe and learn from others in their community yet (Silva et al., 2023). Lastly, language barriers and additional cultural differences could also be at play as foreign-born parents whose primary language is Spanish may struggle to understand the terminology and concepts related to suicide prevention. Such struggles could lead to lower confidence in performing direct suicide prevention tasks (Clay, 2018).

Given that study findings continue to reinforce the importance of direct communication in suicide prevention, particularly among adolescents, providers must consider Latino parents’ comfort. Research by King et al. (2024) identified specific 24-h warning signs for adolescent suicide attempts, emphasizing the need for parents and caregivers to be vigilant and proactive in recognizing these signs. These warning signs include suicidal communications, withdrawal from social activities, and significant changes in behavior or mood. By being aware of these indicators, parents can intervene early and potentially prevent a suicide attempt.

### Study limitations and future research

As with many survey studies, this project has some methodological limitations that warrant a careful interpretation of its findings. The sampling approach, which relied on self-selected participants through Qualtrics panels and social media, introduces potential selection bias that may overrepresent digitally engaged, higher-SES Latino parents—particularly evident in the educational disparities between language groups. The cross-sectional design captures a moment in time and, therefore, fails to capture the dynamic nature of parental self-efficacy, which likely fluctuates with contextual factors and experiences. Perhaps most critically, the emergence of distinct factor structures between language versions (two-dimensional in English versus three-factor in Spanish) potentially challenges the construct equivalence across cultural contexts, undermining direct comparisons between foreign-born and U.S.-born samples if used to do so. As discussed, the reliance on self-reported measures without behavioral validation introduces social desirability bias, especially concerning sensitive topics like suicide prevention. Moreover, language fluency was also self-reported via binary screener questions and was not formally assessed, which may have influenced participants’ comprehension and responses. Finally, the binary categorization of participants as “foreign-born” or “U.S.-born” may oversimplify the complex spectrum of acculturation, potentially obscuring important within-group variations related to immigration timing, country of origin, and other sociodemographic factors that might significantly influence parental self-efficacy beliefs regarding suicide prevention communication.

Future research should incorporate a qualitative approach to better understand how Latino parents conceptualize the questions on the PSPCSE-Lat scales. For example, conducting in-depth interviews or focus groups with parents can provide valuable insights into their thought processes and interpretations of the items. This qualitative data can help refine the scale to better align with the cultural and linguistic nuances of the target population. Recent studies using body mapping have begun to unearth specific body-focused distress signs associated with Latina youth right before a suicide crisis (Gulbas et al., 2025). These signs include physical sensations and emotional manifestations of distress, such as disconnecting, shaking, and heart racing. However, the PSPCSE-Lat-S scale does not currently include items focused on Latino parents’ self-efficacy in identifying such culturally specific embodied suicide-distress warning signs. Incorporating items that address these unique indicators could enhance the scale’s relevance and effectiveness in suicide prevention. Future research should also evaluate the likelihood of parents engaging in conversations about suicide with their children experimentally. This evaluation could be done using interactive virtual reality to simulate parent–child interactions and behaviorally validate the scale’s use. By assessing how parents apply their self-efficacy in real-life scenarios, researchers can determine the practical utility of the PSPCSE-Lat scales and identify areas for improvement.

## Conclusion

This study is significant in that it addresses a gap in research examining suicide-related prevention communication self-efficacy among Latino parents of adolescents in the U.S. while also taking into account this population’s diversity. First, the PSPCSE-Lat-E and PSPCSE-Lat-S scales provide a way to obtain a baseline of Latino parents’ suicide-related prevention communication self-efficacy. The validated scales will offer researchers and practitioners a valuable tool for assessing and enhancing the self-efficacy of Latino parents, ultimately contributing to more effective adolescent suicide prevention efforts within this community. Second, the differing factor structures between the English and Spanish versions shed light on the various ways Latino parents may conceptualize suicide prevention based on their birth origin. Clinicians and prevention specialists should consider such differences and tailor engagement, conversations, and safety planning accordingly. Lastly, the findings highlight differences in comfort with specific prevention tasks that need to be considered when working with the parents of Latino youth who are at risk for suicide, especially when attempting to engage and involve their parents in safety planning.

## Data Availability

The raw data supporting the conclusions of this article will be made available by the authors, without undue reservation.

## Footnotes

^1^For the purpose of this study, biological parents, legal guardians, and caregivers who assume responsibility for childrearing are considered parents for the purpose of this research.

^2^For the purposes of this research, “foreign-born Latinos” are individuals born in the Spanish or Portuguese speaking countries of Mexico, the Caribbean, South and Central America, as well as U.S. citizens born on the culturally- and linguistically-Latino U.S. territory of Puerto Rico.
